# Avoidant Restrictive Food Intake Disorder: A Narrative Review of Types and Characteristics of Therapeutic Interventions

**DOI:** 10.3390/children10081297

**Published:** 2023-07-28

**Authors:** Marcella Di Cara, Chiara Rizzo, Francesco Corallo, Davide Cardile, Rocco Salvatore Calabrò, Angelo Quartarone, Martina Buda, Francesca Cucinotta

**Affiliations:** IRCCS Centro Neurolesi Bonino-Pulejo, S.S. 113 Via Palermo, C.da Casazza, 98124 Messina, Italy; marcella.dicara@irccsme.it (M.D.C.); francesco.corallo@irccsme.it (F.C.); roccos.calabro@irccsme.it (R.S.C.); angelo.quartarone@irccsme.it (A.Q.); budamartina3@gmail.com (M.B.); francesca.cucinotta@irccsme.it (F.C.)

**Keywords:** ARFID, avoidant/restrictive food intake disorder, eating disorders, therapy, rehabilitation

## Abstract

The diagnosis of avoidant/restrictive food intake disorder (ARFID) was added to the diagnostic and statistical manual of mental disorders (DSM-5) just 10 years ago. This disorder consists of the failure to meet one’s nutritional and/or energy needs, which may result in significant weight loss, significant nutritional deficit or functioning dependent on enteral nutrition or oral supplements. In children with this disorder, development is often problematic, and there is also marked interference with psychosocial functioning at all ages. The causes leading to food avoidance in these patients may be related to a lack of interest, to the sensory properties of the food or to the possible adverse consequences associated with it. Given the multitude of aspects involved in this disorder and the impact it has especially on younger patients, more and more studies are addressing treatments and related benefits and/or complications. A narrative review of currently published studies was performed for articles published before 5 March 2023 on therapeutic interventions in patients with ARFID. Because of the large number of results obtained, this review was conducted only via PubMed in order to analyze and discuss children and adolescent ARFID treatments reported in literature. The treatments most often referred to in the literature are cognitive behavioral therapy, family-based therapy and pharmacological treatment. All the data on these treatments are promising. However, due to the recent introduction of this disorder and the limited data still available, a multidisciplinary approach seems to be the best option.

## 1. Introduction

Eating disorders (ED) are a group of mental health conditions that are characterized by disordered eating behaviors and distorted attitudes towards food, weight, and body shape [1]. These disorders tend to resist treatment and have a chronic and disabling course [2]. The World Health Organization identifies them as the second leading cause of death among adolescents after road accidents. The most common types of ED include Anorexia Nervosa (AN), Bulimia Nervosa (BN) and Binge Eating Disorder (BED). In AN, the patient has a distorted body image and an intense fear of gaining weight, and restricts his food intake, often to the point of starvation, and a significantly low body weight [3]. Patients with BN engage in binge eating followed by purging behaviors such as vomiting or using laxatives to rid themselves of the food they have consumed. They also have a distorted body image and an intense fear of gaining weight. BED is characterized by episodes of binge eating where individuals consume a large amount of food in a short period of time and feel a loss of control over their eating behavior [4,5]. They may also experience feelings of guilt or shame after a binge. These disorders can have serious physical and psychological consequences, including malnutrition, gastrointestinal problems, electrolyte imbalances, depression, anxiety, and social isolation [6]. Because of this, medical associations in the USA that deal with these disorders describe them as a real epidemic that cuts across all social strata and ethnic groups [7]. Although they have a different nature, they share a common psychopathological core: an excessive and altered focus on body shape on which the subject’s self-esteem depends. Causes may include environmental, genetic, psychological, or socio-cultural factors. This multifactorial etiopathogenesis is reflected in the uniqueness with which these disorders present themselves in each individual [2].

Although the main features of the disease had already been described earlier, only in 2013 was the diagnosis of Avoidant/Restrictive Food Intake Disorder (ARFID) introduced in the DSM-5 [8]. ARFID is characterized by a persistent failure to meet appropriate nutritional and/or energy needs, which leads to one or more of the following:

- Avoidance or restriction of food intake based on the food’s sensory characteristics (e.g., texture, smell, taste);
- Lack of interest in eating or food;
- Fear/concerns of adverse consequences of eating, such as choking or vomiting, gaining weight, or developing an illness.

Unlike other ED, such as BN and AN, ARFID subjects do not have distorted body image or the desire to lose weight. Rather, they experience anxiety or distress related to eating or certain foods, which can lead to a significant decrease in food intake and associated nutritional deficiencies, impaired growth, and psychosocial impairment [9]. ARFID replaces and extends the DSM-IV diagnosis of childhood ED. In fact, it is the most recent ED diagnosis included in the DSM-5. The range of restrictive eating behaviors present throughout life are not motivated by an intense fear of gaining weight or one’s physical fitness. These behaviors are often described as “selective eating” or as food phobias. Many of these, particularly those with selective eating, have long been recognized and treated within the medical community under the title of ED [10]. This is a common problem in childhood, with between 13 and 22 percent of children aged 3 to 11 years being reported as “picky eaters” [11]. Mascola et al. [12] in their study showed that between 18 and 40 percent of food-related rigidity tends to persist into adolescence.

Avoidance, disinterest, or careful selection of food, typical of patients with ARFID, is often mistaken for a tantrum. In order to be able to distinguish a picky child from a subject with ARFID, the frequency of episodes and caloric intake must be assessed: if they are sporadic and if they do not reduce caloric intake below daily requirements, they may be stand-alone and not pathognomonic.

The onset of the disorder occurs in childhood or early adolescence (in some cases in adulthood). As for the average age of onset, it is believed to be earlier than in other ED, with onset often occurring between the ages of 9 and 12 years, regardless of culture or social status. There are not yet many epidemiological studies in the literature, however, the disorder would seem to predominantly affect males, who are also those in whom there is greater selective eating, and a high degree of concomitant anxiety [13]. Approximately 15 percent of patients who seek treatment at specialized centers meet the criteria for ARFID and have an average weight of 85 percent of expected weight, compared with 81 percent in AN and 107.5 percent in BN [14]. A multicenter study found a 14% prevalence of ARFID diagnosis among adolescents and children who present themselves as new patients to medicine programs [15,16].

ARFID can occur at any age, but it is most commonly diagnosed in childhood or adolescence. It can be provoked by several factors, including sensory sensitivity, developmental disorders (such as autism spectrum disorder), trauma, anxiety, or a history of medical conditions that have made eating difficult or painful [17]. 

The etiology of this disorder is currently unknown. Thomas et al. [18], in their three-dimensional model, hypothesize the existence of a genetic predisposition leading to abnormalities in taste perception and homeostatic appetite. Traumatic experiences related to food could trigger this predisposition, causing a restriction or avoidance of food intake. However, the model remains hypothetical as it lacks specific biological markers, and thus empirical validation.

Environmentally, an anxious parental style and the presence of ED in parents seem more likely to predispose to the onset of the disorder [8]. Misinformation toward certain foods/eating styles and the subject’s traumatic experiences—such as having a risk of choking or the first time vomiting—may influence preference or avoidance toward certain foods.

The psychological aspect also plays a key role. Usually in fact, individuals with ARFID show difficulties on an emotional level such as sadness, anxiety, and concern about food and eating. These emotions cause them to manifest a disinterest in eating. These children struggle to perceive the sense of hunger and often forget to eat. When they do eat, they report feeling full early. Children who manifest some degree of opposition toward food are more likely to experience emotional and behavioral changes, such as depression, generalized anxiety, or more specific ones such as fear of choking from an event that has previously frightened the child [19,20,21]. They may be children who exhibit irritability, distractibility, and restlessness not only at mealtime.

Given the complexity and recent introduction of this disorder, this paper aims to analyze and synthesize existing literature on ARFID treatments in children and adolescents. Since a gold standard for the treatment of ARFID pathology has not yet been established, this study could allow the confrontation between the characteristics and effects of different interventions and suggest areas for further investigation. The narrative review was conducted to ensure an inclusive synthesis of evidence and to offer more robust conclusions applicable to different populations.

## 2. Materials and Methods

A narrative review of currently published studies was performed for articles published before 5 March 2023 on therapeutic interventions in patients with ARFID. Due to the large number of results obtained, the search was conducted exclusively through PubMed. Other databases were not taken into consideration. Research was conducted using the following search keywords and terms: ((arfid) OR (avoidant food intake disorder)) AND (therapy); ((arfid) OR (avoidant food intake disorder)) AND (treatment); ((arfid) OR (restrictive food intake disorder)) AND (therapy); ((arfid) OR (restrictive food intake disorder)) AND (treatment).

### 2.1. Inclusion Criteria 

A study was included if it described or investigated the effects and/or medical, pharmaceutical or psychological interventions in children and/or adolescents with ARFID. Only articles written in Italian or English were included in the review. 

### 2.2. Exclusion Criteria

A study was excluded if there was a lack of ARFID treatment models or if it involved animal and/or cellular models. Systematic, integrative, or narrative review were also excluded, although their reference lists were checked and included if appropriate. All articles written in languages other than Italian or English were excluded. No restriction due to the year of publication was adopted since the recent development of ARFID.

## 3. Results

**Figure 1:**
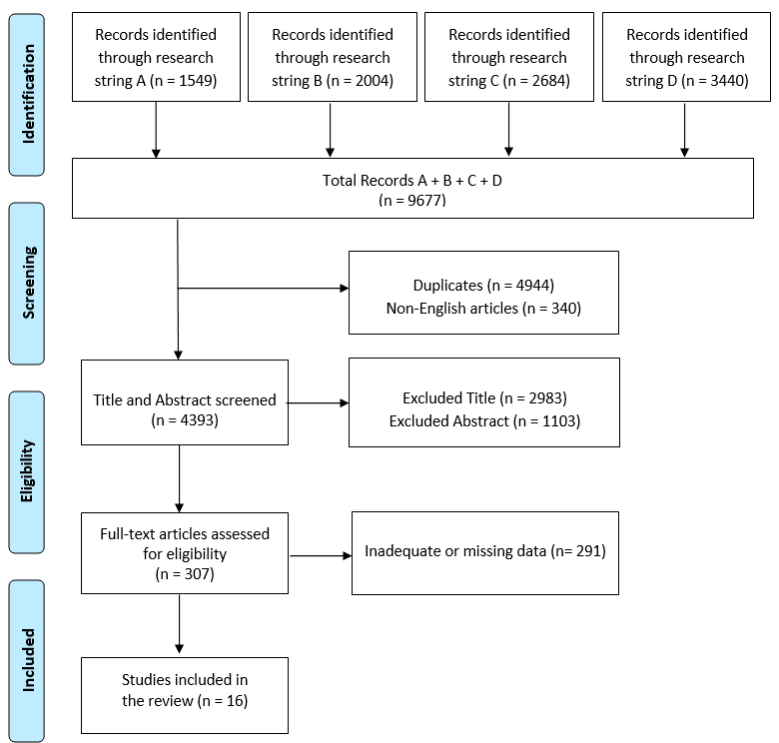
PRISMA flow chart for the current review.

The initial electronic data search yielded a total of 9677 potentially relevant studies on PubMed. Of these, 4944 were duplicated and 340 were non-English articles. 4086 articles were excluded due to title or abstract (Figure 1). 

Of the resulting 307 articles, 16 fully met the inclusion criteria and were therefore included in the review. All articles delving into different types of treatment: hospital treatment [22,23], cognitive behavioral-therapy [24,25,26,27,28,29], family-based treatment [30,31,32,33], and two or more treatments [34,35,36,37]. A summary of these studies is shown in Table 1.

### 3.1. Hospitalization and NGT Feeding

Treatment for Avoidant Restrictive Food Intake Disorder (ARFID) varies from multidisciplinary outpatient team treatment to medical hospitalization [23,34,35,36,37]. As ARFID has recently been recognized as a distinct disorder, limited evidence-based guidelines currently support treatment strategies [22]. A medical team comprising a physician and mental health specialist, along with additional healthcare professionals such as a dietician, gastroenterologist, occupational therapist, and/or speech therapist, should be involved based on the patient’s specific needs. Given the scarcity of ARFID evidence, treatment objectives can be set similar to other restrictive eating disorders, such as restoring weight and promoting menstruation in amenorrheic women [35]. ARFID exhibits phenotypic variations, necessitating tailored interventions to address diverse presentations. Psychosocial interventions, such as psychoeducation on ARFID, exposure, habituation, and nutrition, have shown significance [28]. Involvement of family/caregivers is crucial, especially for younger patients [30,31,32,33]. Therapeutic approaches involving in/out-session exposure work and organized meals have been widely adopted [29]. Additional methods include the use of reinforcers for behavior changes, sensory and self-regulation treatments, management of comorbidities, pharmacotherapy (e.g., cyproheptadine or mirtazapine to stimulate appetite), and further medical treatment [22,27,35,37].

Certain ARFID patients may require hospitalization due to underlying medical conditions [23,38]. Guidelines for hospitalizing individuals with restrictive eating disorders have been outlined [39]. ARFID patients may reach a state of homeostasis, exhibiting less hypotension and bradycardia compared to those with Anorexia Nervosa (AN) who are still losing weight [40]. Medical intake, based on the patient’s BMI compared to the median BMI for their age and sex, can help determine if hospitalization is necessary. Structured re-feeding protocols can aid weight increase and monitor electrolyte changes to prevent re-feeding syndrome [14,41]. As ARFID patients may struggle with volume and variety, facilitating an initial increase in volume with preferred foods may be beneficial [42]. ARFID patients hospitalized for eating disorders tend to rely more on enteral nutrition than subjects with AN [43,44,45,46,47]. Deciding between supplement use or solely relying on food intake should consider factors like dietary intake, motivation, and restrictions [48]. ARFID patients may have a history of long-term enteral feeding in outpatient settings, contributing to the higher frequency of tube feeding [49]. While tube feeding is crucial in acute malnutrition cases, its use should be provisional, with the goal of achieving oral intake for appropriate nutrition. Weaning from tube feeding is usually administered under close supervision in an inpatient or day treatment setting once a healthy weight and oral nutrition tolerance are achieved. Healthcare providers should assess if outpatient psychotherapy is sufficient or if more intensive treatment programs, like day treatment or intensive outpatient programs, are necessary [24].

ARFID often leads to nutritional shortcomings of varying severity and type, affecting manifestations of the disorder. Prolonged and inadequate food intake can lead to adaptive nutrient conservation, masking laboratory abnormalities. Therefore, clinical assessment should consider dietary intake and clinical presentation, not solely rely on lab values [50]. Timely replenishment of nutrients like vitamin B12, vitamin C, iron, zinc, and folate are crucial to prevent negative effects on taste, hunger, mood, and energy. These deficiencies can hinder treatment adherence, necessitating timely addressing [50]. However, high initial doses of nutrients are difficult to achieve through diet alone, requiring prolonged supplementation to effectively reverse deficiencies. Alongside supplementation, encouraging dietary intake rich in deficient nutrients is essential for health and satiety. Clinicians should monitor lab values to assess supplementation effectiveness and adjust as needed.

Cyproheptadine, an antihistamine, has been used off-label to treat ARFID patients with food disinterest [51]. By blocking serotonin receptors in the brain, cyproheptadine can increase appetite and improve gastric accommodation. Studies in children with feeding difficulties showed higher improvements in weight gain and eating behavior with cyproheptadine [35,37]. Not all ARFID patients benefit from cyproheptadine, and tolerance may develop over time, requiring temporary discontinuation [35,37]. Its use should be supervised by medical professionals, considering potential risks and benefits. Addressing underlying psychological and behavioral factors is essential in a comprehensive treatment plan. Overall, comprehensive, individualized treatment plans considering psychosocial, medical, and dietary aspects are necessary to effectively manage ARFID and improve patient outcomes.

### 3.2. Cognitive-Behavioral Treatment

In the cognitive-behavioral approach, classic cognitive treatment (i.e., the analysis and refutation of dysfunctional thoughts) remains central and is supplemented by contributions from behaviorism and functional analysis [27]. Thoughts, emotions, and behavior influence each other. Negative dysfunctional thoughts can influence behavioral aspects of the individual. Similarly, behavior can reinforce certain negative thoughts, help the patient recognize these self-sustaining mechanisms, and act to change them [24]. Treatment moves both on a cognitive level (with cognitive analysis and restructuring) and on a behavioral level (with behavioral prescriptions [25,26,28,52,53]. The ultimate aim is to go on modifying the patient’s maladaptive deep-seated beliefs and thus favor lasting change [27]. To achieve this, CBT uses various techniques. Psychoeducation consists of actively informing patients about their psychiatric disease with the aim of helping them change the dysfunctional cognitions and behaviors that retain their disorders [54,55,56,57]. This technique has proven useful in handling many other psychiatric disorders, including depression [58], and has also been shown to be valid for populations with ED [59,60].

Another behavioral technique is the systematic desensitization [61]. The stimulus that causes anxiety in patients is recognized, and a hierarchy of different forms of the stimulus ordered from weakest to strongest is identified. In the case of an ED, the patient formulates a fear/anxiety hierarchy of safe and unsafe foods [28,62,63,64]. Next, the subject is instructed in techniques for coping with anxiety, such as muscle relaxation. By sequentially relaxing different muscles of the body (legs, arms, head, neck, shoulders, chest and stomach), an increasingly deeper level of relaxation is achieved [58]. Finally, the subject implements the learned techniques towards the stimulus causing anxiety or phobia, proceeding according to the hierarchy initially identified [28,65,66]. Generally, one moves from even imaginary exposure to an in vivo but controlled exposure.

Cognitive restructuring challenges the patient’s thoughts by trying to grasp their logicality, validity, or function [25,67]. The aim is to replace irrational thoughts with more functional ones. Automatic thoughts often activate a state of anxiety or catastrophic beliefs in the patient that alerts him/her to situations perceived as potentially dangerous [68]. In this state the patient imagines that he or she is overwhelmed by the event because of its dangerousness or because of his or her own inability to cope with it [69]. 

The Enhanced Cognitive-Behavior Therapy is a treatment approach developed by Calugi et al. [70,71] to address the core psychological issues underlying all types of ED. CBT-E operates under the assumption that the eating issue is specific to the subject and encourages adolescents to take control of their own recovery. While parents can participate in the care process, their inclusion is limited to fostering the creation of a supportive family setting. Patients are active participants in all stages of treatment and proactive changes are promoted. CBT-E is a collaborative approach that aims to assess the patient’s ED, including concerns about weight, shape, dietary restraint, and further extreme control behaviors [34].

Even more specific is the cognitive-behavioral therapy for ARFID (CBT-AR), a flexible treatment that generally comprises 20–30 sessions. Initially, the therapist provides psychoeducation on ARFID, spurs patient to eat regularly and to self-control, and depending on the variety of the disorder helps the patient to increase food variety and/or quantity [72,73]. Subsequently, psychoeducation is administered on nutritional deficiencies and the patient is supported and encouraged in the selection of new foods that resolve these deficiencies and/or reduce the clinical impairment. The therapist also takes care of selecting suitable modules for the patient’s ARFID maintenance mechanisms. Interventions obviously differ depending on the module, but common elements include in-session exposure and practice among sessions. Finally, progress is evaluated by the therapist who creates a relapse prevention plan [16]. 

### 3.3. Family-Based Treatment and Parental Training

Family-Based Treatment (FBT), also known as the “Maudsley approach”, is a therapy approach for ED that involves the whole family in the treatment process [74]. FBT was initially developed for the treatment of AN in adolescents, but it has since been adapted for other ED, such as bulimia nervosa and binge-eating disorder. In a number of clinical cases published recently, family-based treatment (FBT) has been used to treat adolescents and children with ARFID [30,31,32,33]. FBT for ARFID is similar to FBT for anorexia nervosa in that parents take responsibility for feeding their child but differs from FBT for AN in that parents are encouraged to help their child increase not only the quantity, but also the diversity of the food they consume, through repeated exposure to new foods [75,76]. 

In FBT, the family is viewed as a resource for helping the individual with the ED. The therapy is typically divided into three phases. In the first phase, the focus is on restoring the individual’s weight to a healthy level. The second phase involves gradually transferring control of eating from the parents to the individual with the ED. The final phase focuses on addressing broader family issues that may have contributed to the development or maintenance of the ED [77]. FBT typically involves weekly family therapy sessions with a trained therapist, as well as individual sessions with the individual with the ED. The therapy also involves regular weighing and monitoring of the individual’s food intake [78,79].

Le Grange et al. [34] confronted the relative efficacy of FBT and CBT-E. Their results show that FBT is effective to increase weight gain among underweight adolescents similarly to CBT-E. Lock and colleagues [30] provided three brief case reports that exemplify the application of FBT in the process of care of pre-adolescents diagnosed with ARFID. The authors’ work demonstrates how family-based treatment (FBT) can effectively address three different clinical presentations of ARFID: low appetite and lack of interest, sensory sensitivity, and fear of negative consequences. Their findings suggest that FBT’s fundamental principles, such as agnosticism towards the cause of the externalization, illness, emphasizing ARFID severity, empowering parents, providing behavioral consultation, and offering practical attention to behavior, can be applied to a variety of ARFID presentations. However, treating children with ARFID using FBT may involve common challenges, such as promoting urgency, facing long-term behavioral accommodations, addressing lack of parental alignment or fatigue, and managing comorbid psychiatric issues in patients. Overall, the authors propose that FBT can be adapted to effectively treat children with ARFID by applying its core principles.

### 3.4. Pharmacological Treatment

There are no established guidelines from a psychopharmacological perspective for treating ARFID. However, as reported by the World Federation of Societies of Biological Psychiatry (WFSBP) guidelines on the pharmacological treatment of eating disorders [74] the initial aim is usually to restore the patient’s healthy weight and alleviate the stress and anxiety associated with eating. Medications can be used to target specific symptoms, such as severe situational anxiety about eating, decreased appetite, or premature satiety resulting from chronic malnutrition [18]. However, it’s crucial to carefully consider the use of pharmacological agents and combine them with other therapeutic approaches, including behavioral treatment and multidisciplinary interventions like FBT. Benzodiazepines, like Lorazepam, may be suitable for short-term use in extremely tense patients, especially when implementing meal plans. Cyproheptadine is a safe and effective option for children with feeding difficulties related to low appetite [10] while low-dose Olanzapine can reduce anxiety, stimulate appetite, and facilitate eating. D-Cycloserine has also been shown to be beneficial in exposure interventions for anxiety disorders [80].

Selective serotonin reuptake inhibitors, such as fluoxetine and sertraline, are typically the first-line treatment for anxiety and depression in pediatric patients, although their side effects (nausea, vomiting, reduced appetite) can worsen feeding problems in patients with ARFID. In this case, mirtazapine was chosen as a pharmacological adjunct because of its numerous advantages, including alleviating anxiety and sleep issues, reducing nausea, increasing appetite, and improving stomach emptying.

### 3.5. Telemedicine 

Telemedicine emerges as a desirable method for feeding assessment and therapy as it allows useful home environment observations, including interactions with parents that may influence feeding issues, thereby improving generalization to the home setting [81]. The term “Telemedicine” (TM) refers to a delivery modality for health care services which occurs through the use of communication technologies. When the physician and patient are distant, they can make use of telemedicine [82]. Since the process may also involve at-home patient self-care and self-monitoring regimens, the term TM is used as a synonym for “telehealth” and “telecare” [83]. Therefore, it constitutes a treatment modality rather than a treatment itself [84]. Bloomfield et al. [29] in their study highlight the potential benefits of using teleconsultation to implement a behavioral feeding intervention in the treatment of ARFID. Their results show that telemedicine has a high level of family compliance and acceptability, and that it is effective in increasing food variety in a child with ARFID. Telemedicine is perceived as a service that makes it easier to start treatment for the patient [85,86] especially for people who live in rural sites [87]. For families, it allows them to cut travelling and waiting time, saving money [85] and easily accessing specialist care [88]. Research has demonstrated positive results when utilizing video conferencing as a means of providing therapy for children and teenagers dealing with chronic illnesses [89] or depression [90]. Furthermore, an 8-week teleconsultation program for parents of children with ADHD showed promising outcomes, with a reduction in parental stress and improvement in the children’s behavior [91]. Reports from parents who have participated in teleconsultation interventions have been overwhelmingly positive, indicating high levels of satisfaction with the treatment [92].

## 4. Discussions and Conclusions

The introduction of the ARFID diagnosis in the DSM-5 was a significant advancement in achieving better diagnostic consistency and recognition of patients who had previously been marginalized in clinical settings. ARFID is a complex disorder with various presentations and aspects, making its diagnosis and treatment a multidisciplinary issue. The treatment modalities for ARFID include pediatric, behavioral, dietary, mental health, individual, family, and group therapy, along with psychopharmacology in some cases. CBT-based approaches [24,25,26,27,28,29] were found to be effective in decreasing symptom severity, food phobia levels, and family accommodation [25,26]. Moreover, these treatments have been shown to be effective in increasing food variety [24] and achieving a healthy body weight and nutritional intake [26]. Many other studies confirm these results [10,93,94], and found improvement even in psychosocial functioning. While interoceptive exposure could decrease ARFID symptoms and increase general self-regulation skills [28], extinction-based treatment and exposure to disgust treatment should still be further investigated [95,96].

Studies that have investigated the effects of family-based treatment have found that it is applicable to various clinical presentations of ARFID with a positive effect on symptoms and weight gain [30,31,32,33]. FBT is also highly acceptable for families and better than usual care [34]. FBT has been identified as a promising area to investigate further in the ARFID context [97]. Le Grange et al. [34] in their work made a comparison between CBT-E and FBT and found that FBT improved weight gain better than CBT-E, but not at follow-up. However, Rienecke et al. [98] state that different ARFID subtypes benefit more from different strategies. It seems that patients with limited intake benefit most from FBT, while aversive patients benefit most from CBT for anxiety. Finally, patients with a limited variety of foods benefit more from multidisciplinary behavioral interventions. 

There are currently no specific pharmacological treatments approved for ED. The only exception is fluoxetine for bulimia nervosa. ARFID patients are treated with drugs to treat diseases often concomitant with the disease or are treated with drugs approved for other disorders but which have been shown to have positive effects on ED [35]. Dolman et al. [37] for example, in their work, treated a young patient with olanzepine and sertraline in order to manage anxiety, nausea, and to stimulate appetite. The authors have combined this pharmacological treatment with CBT and FBT with good results [37]. D-Cycloserine has been shown to be a valid option in interventions for anxiety disorders [74]. In the case of aversive subtype patients, fluoxetine, paroxetine, escitalopram, sertraline, and fluvoxamine were shown to be beneficial [98,99,100,101].

Since the drug was also found to be functional in other studies [18,102], Naviaux et al. [35], in their work, successfully used mirtazapine for treatment. It was found that this drug promotes weight gain and appetite, decreases nausea, and enhances gastric emptying [103]. This occurs because Mirtazapine increases noradrenergic and serotoninergic neurotransmission [104]. Mirtazapine has been found to alleviate anxiety and sleep issues [105]. Many studies [106] also report Cyproheptadine as a valid drug to stimulate appetite. 

The practical implications that this study drew from the evidence in the literature are described as follows. A nasogastric tube feeding or an enteral feeding may be a necessary strategy in the treatment of acute malnutrition, especially in cases involving severely underweight patients. However, due to the oral sensitivities of ARFID patients and the potential psychological consequences, its use has to be considered more carefully [107]. Experience of NGT feedings could be particularly aversive and create iatrogenic conditioned food aversions [108]. Once patients have reached an adequate weight and are able to take most of their nutrition orally, NGT must be suspended.

Some of the treatments discussed so far can be administered using telemedicine technologies. Bloomfield et al. showed that the use of teleconsultations for implementations of CBT results in an increase in food variety in an ARFID child [29]. This modality was perceived as highly acceptable by patients’ families and simplifies the start of treatment and access to specialist care [87] for patients who have limited mobility or who live in rural sites [86]. Telemedicine represents an opportunity, especially for patients who have limited mobility or live in rural areas. It allows for cutting travelling and waiting time, saving money, and increasing families’ satisfaction. 

Among the limitations of this study, it should be noted that due to the large number of results obtained from the search strings, only one database (Pubmed) was used. In addition, perhaps the choice of more databases and the use of a better search string would have allowed for a more specific yet more thorough investigation. Due to its multifaceted nature, ARFID is a complex disorder that requires polytherapeutic treatment involving a multidisciplinary team working and collaborating for the well-being of the patient. ARFID’s various presentations and comorbidity may require BCT, a dietetic approach, and FBT. In some cases, psychopharmacology can be very useful but should be carefully considered. Nutrition via a nasogastric tube must also be closely considered because it could give rise to negative iatrogenic effects that would adversely affect the course of treatment.

A multidimensional approach proves to be the best choice, as it allows for due consideration to be given to many aspects of the disease that affect not only the patient, but also the family.

## Figures and Tables

**Table 1:** Summary of studies included in the research.

Study	Aim	Method	Treatment Modality, Period and Recipients	Clinical Outcome
Shimshoni et al., 2020 [22]	To assess the feasibility, acceptability, and preliminary effectiveness of Supportive Parenting for Anxious Childhood Emotions adapted for ARFID (SPACE-ARFID).	Family accommodation and ARFID severity were evaluated before and after the treatment in 15 families. Satisfaction was evaluated.	Hospital treatment 12 sessions Children	ARFID-related interference ratings and symptom severity were significantly reduced. 13 participants added from 1 to 14 new food/beverages in their diet. Food-related flexibility increased, while family accommodation decreased after the treatment. Satisfaction levels were high both in family and patients.
Bryson et al., 2018 [23]	To evaluate the long-term effects of the partial hospitalization program for ED on ARFID patients.	ARFID patients were compared with AN patients who had been discharged at least 12 months previously. Medical record data, Children’s Eating Attitudes Test (ChEAT) scores, and median BMI, were evaluated.	Hospital treatment 7 sessions. Children and Adolescents	AN and ARFID patients showed similar BMI increases from intake to discharge, whereas in ChEAT they showed low scores at discharge. Both patient types maintained low scores in both ChEAT and weight at subsequent follow-ups. Compared to AN patients, there were fewer ARFID patients receiving post-treatment care
Thomas et al., 2020 [24]	To assess acceptability, proof of concept and feasibility of CBT in ARFID patients (CBT-AR).	CBT-AR was offered both to patients in individual or family format.	CBT 20–30 sessions. Children and Adolescents	The subgroup composed of underweight patient moved from the 10th to the 20th BMI percentile showing significant weight gain. Patients introduced a mean of 16.7 (SD = 12.1) new foods. ARFID severity decreased and 70% no longer met criteria for the pathology.
King et al., 2015 [25]	To illustrate the case of an ARFID patient with anxiety disorder treated with CBT.	Cognitive and behavioral techniques, specifically psychoeducation, systematic desensitization and cognitive restructuring were employed.	CBT. 6 sessions. Adolescent	Eating meal percentage augmented to an interval between 70–100%. Anxiety resulted to be reduced and BMI increased to 17.4 kg/m^2^.
Dumont et al., 2019 [26]	To test the functionality of a new exposure-based daytime CBT treatment in ARFID patients.	ARFID patients (10–18 years) underwent four weeks of CBT. At baseline and at 3 month follow up food selectivity test, a 1-week food diary and behavioral measures of food intake were performed.	CBT. 28 sessions. Children and Adolescents	At the end of the treatment food acceptance increased in 91% of patients, and 6/11 patients did not meet any of ARFID criteria anymore. At follow-up all but one of patients were in remission. Food neophobia score decreases as well as anxiety levels. Food frequency and intake also increased, indeed gained weight, and achieved an adequate nutritional intake for their age.
Taylor et al., 2021 [27]	Assess whether the benefits of the treatment can be retained when parents are trained and continue the program at home and during meals outside the home.	26 ARFID children were offered. The parents were trained so they could continue it at home. Treatment outcomes included mealtime behavior and the range/quantity of food eaten.	CBT. 11 sessions. Children	All therapeutic goals agreed upon at the beginning of the treatment were achieved by the patients. On average, the patients introduced 92 different foods into their diet. Improvements were recorded at the end of treatment and were maintained at subsequent follow-ups up to an average of 2.3 years. Parents’ levels of acceptability and satisfaction with the treatment were high.
Zucker et al., 2019 [28]	To present the case of a 4 years old ARFID patient to enhance knowledge about the treatment efficacy.	Patient was treated by Feeling and Body Investigators -ARFID Division, an interoceptive exposure treatment based on acceptance. Using playful cartoons and other exposures, patients were taught to recognize their feelings, emotions, and actions and to give them meaning.	CBT. 11–15 sessions. Children	Interoceptive Exposures revealed themselves as an effective method that increases self-regulation abilities and reduces ARFID symptoms. Patient reported to enjoy the treatment. Quantity of food improved after two sessions, and spontaneous requesting of snacks increased after six. At the end of treatment, patient no longer met criteria for ARFID. Monthly maintenance therapy was continued at parents’ request.
Bloomfield et al., 2018 [29]	To examine the effectiveness of teleconsultation in implementing a behavioral intervention to increase the variety of foods in an ARFID patient.	Target behavior was consumption of 10 bites of a nonpreferred food. Reinforcements were placed on a fixed ratio. Patient received reinforcement following every successful bite consumed without expelling the food.	CBT. 12 sessions Children	Bites frequency for non-preferred foods increased following successive boosts in criteria. Acceptance level for the technology process and intervention was high.
Lock et al., 2018 [30]	Describe how to use FBT in the treatment of ARFID patients.	The use of FBT in patients without weight problems with three different clinical presentations of ARFID was analyzed: (1) lack of interest and poor appetite; (2) eaters sensitive to the physical characteristics of food and (3) eaters fearful of adverse effects.	FBT. 17–19 sessions. Children and Adolescents	There was consistent weight gain and eating related cognitions improvement. Mental health conditions also resulted to be improved.
Lock et al., 2019 [31]	To assess modifications between FBT treatment and usual care (UC).	Effect size (ES) differences between the two approaches were evaluated.	FBT. Unspecified. Children and Adolescents	For clinical severity and weights measure ES differences were moderate to large, favoring FBT-ARFID over UC. Both normal weight and underweight patients gained more weight in FBT-ARFID and severity of symptoms resulted reduced. FBT-ARFID resulted to be acceptable to families and preferable even by improvement recorded.
Spettigue et al., 2018 [32]	Illustrate the clinical cases, treatments, and outcomes of 6 arfid patients in the context of a hospital ED program.	Treatment included family therapy, CBT, drug treatment (olanzapine, fluoxetine, cyproheptadine) and monitoring.	FBT. 14–28 sessions. Children and Adolescents	All patients achieved their goal weight, reducing tantrums and augmenting food intake varieties. All patients responded well to treatment, and it reduced their anxiety in general and at school.
Rosania K., Lock J., 2020 [33]	Describe the use of FBT in a 9-year-old patient sensitive to the physical characteristics of food.	9-year-old patient underwent 2 phase FBT.	FBT. 17 sessions. Children	At the end of treatment, the patient gained 2.1 kg, the severity of clinical symptoms decreased to the point where she no longer met the DSM criteria for ARFID. Patient introduced 13 new foods in her diet.
Le Grange et al., 2020 [34]	To make a comparison between the effectiveness of FBT, CBT-E.	107 patients diagnosed with an ED chosen between CBT-E and FBT. Evaluations were conducted before and after treatment, 6 and 12 months later.	FBT or CBT. 20–40 sessions. Children	At the end of the treatment weight gain was found to be significantly higher in FBT than in CBT-E, but this did not occur at follow-up. Higher weight gain was achieved for patients with lesser externalizing problems, no psychiatric disorder, and no history of abuse. CBT-E was chosen by less well and older participants.
Naviaux A.F., 2019 [35]	Report an ARFID presentation, identification, and its management modalities in a pediatric ward of a general hospital.	A 12-year-old ARFID patient case, treated by a multidisciplinary team, was described and a literature review was conducted.	FBT and pharmacological treatment. 3 sessions. Children	During three admissions, the patient underwent a partial hospitalization model, FBT and mirtazapine. The treatment was successful. Patient’s quality of sleep, stress and fatigue improves. Nausea disappears and she starts to eat more regularly.
Schermbrucker et al., 2017 [36]	To assess treatment challenges of ARFID and to discuss socio-cultural factors that may contribute to not accepting a psychological diagnosis.	The case of an 11-year-old Colombian ARFID patient was described. Food exposure therapy, group therapy and pharmacological treatment was offered, but patient declined all of them, so NGT feeding was performed. The role that culture plays in the diagnosis of the disease was analyzed.	CBT, pharmacological treatment, NGT. Unspecified Children	After the discharge, patient had a weight of 39.8 kg (97% of his ideal body weight) and his nutritional intake was 3000 kcal per day. The results show that the potential issues that emerged on the cultural side of treatment and diagnosis and the disorder could be useful for both health professionals and the patient.
Dolman et al., 2021 [37]	Describe the case of an 11-year-old ARFID patient with the characteristics of all three ARFID subtypes.	A therapeutic approach that combined aspects of drug therapy (sertraline and olanzapine), CBT, FBT was used.	CBT, FBT, pharmacological treatment. Unspecified Children	Patient achieved a 27% weight gain from admission, and a BMI of 18.9 (>85th percentile for age). Ferritin and hemoglobin levels improved as well as vitamin A, C, and E deficiencies. Patient introduced 3 new foods into the daily diet.

Legend: ARFID = Avoidant Restrictive Food Intake Disorder; ED = Eating Disorder; AN = Anorexia Nervosa; FBT = Family Based Treatment; CBT = Cognitive Behavioral Treatment; CBT-E = Cognitive Behavioral Treatment Enhanced; BMI = Body Mass Index; NGT = Nasogastric tube. ChEAT = Children’s Eating Attitudes Test; SPACE-ARFID = Supportive Parenting for Anxious Childhood Emotions adapted for ARFID; UC = usual care; ES = Effect size.
